# Hard Chancre of Bulbar Conjunctiva of Child of Ten Years

**Published:** 1917-04

**Authors:** 


**Hard Chancre of Bulbar Conjunctiva of Child of Ten Years.** H. Fromaget, Jour. de Med. de Bordeaux, July 1916, reports an ulceration 3x10 m.m. Wassermann reaction positive, negative in mother and sisters. Source of infection not determined. Cure by novarsenobenzol, 0.15 intravenously. This is the third case seen by the author.

